# Resposta a ‘’Reinternação de Pacientes com Síndrome Coronariana Aguda e seus Determinantes’’: Uma Visão da APS

**DOI:** 10.36660/abc.20190817

**Published:** 2020-04-06

**Authors:** Laís Cruz Lima, Eliane Mazzuco, André Luís Prudêncio

**Affiliations:** 1 Universidade do Sul de Santa CatarinaTubarãoSCBrasilUniversidade do Sul de Santa Catarina (UNISUL), Tubarão, SC – Brasil

**Keywords:** Atenção Primária à Saúde, Hospitalização/economia, Readmissão do Paciente/economia, Serviços de Saúde, Síndrome Coronariana Aguda, Fatores Socioeconômicos

O estudo de Oliveira et al.,^1^ mostra dados de suma importância sobre reinternações de pacientes em hospitais em até um ano após internação prévia por SCA e seus determinantes. Nesse estudo, foram avaliadas inúmeras variáveis, e entre as mais determinantes destaca-se o tipo de serviço de saúde utilizado pelos pacientes durante as internações.

Dessa forma, para melhor compreensão da realidade retratada não só na cidade-sede do estudo, mas em inúmeras outras cidades por todo o Brasil, e a fim de permitir maior entendimento sobre as variáveis abordadas, gostaríamos de salientar questões anteriormente discutidas em arsenais literários.

O artigo mostra que, dos 21,46% dos pacientes que necessitaram de reinternação, a maioria utilizava serviços privados de saúde, ressaltando um dos fatores de risco utilizados no presente estudo: o perfil socioeconômico dos participantes. Já entre aqueles que dependiam do sistema público de saúde foi menor a taxa de readmissão, pois, como as vagas são limitadas, nem todos são internados, ao contrário do que se observa no serviço privado.

O estudo afirma que a procura pelo serviço hospitalar ocorre devido à dificuldade de acesso a consultas em nível primário, evidenciando uma falha na comunicação entre a atenção básica à saúde e a população, que, a longo prazo, tem reflexo nas internações hospitalares e seus altos custos, potencialmente evitáveis. Segundo Cecílio et al.,^2^ quando se trata do acesso da população ao serviço de saúde, a atenção básica é a porta de entrada para esse sistema, ressaltando um dos papéis fundamentais da Unidade básica: a prevenção, seja primaria, secundaria ou terciaria.

Para Starfield^3^ a Atenção Básica à Saúde que deve coordenar os fluxos dos usuários entre os vários serviços de saúde, buscando garantir maior equidade no acesso. Todavia, para isso é necessária uma comunidade informada e amparada, que seja incentivada a prevenir doenças, cardiovasculares ou não, tanto antes quanto depois de internações prévias. Essa realidade é possível a partir de campanhas, projetos, rastreamento e acompanhamento feitos pela unidade de saúde responsável em cada região.

Desse modo, é de suma importância que os profissionais de saúde informem a população sobre os serviços oferecidos pela Atenção Básica de Saúde e suas funções em relação a prevenção de doenças e hospitalização. Assim agindo, garantem uma alternativa potencialmente capaz de reduzir as taxas de internação, pelo serviço público de saúde, durante e após a vigência de SCA. Desta forma, será possível fazer jus à aplicação da Lei 8.080,^4^ de 19/9/1990, da Constituição brasileira, que visa à promoção de saúde para todos.

## Carta-resposta

Somos gratos pelos comentários e pelo interesse por nossa pesquisa, que versa sobre um tema relevante, no qual se destaca a reinternação de portadores de síndrome coronariana aguda (SCA) e seus determinantes, em usuários dos serviços de saúde, tanto da rede pública (SUS) como da rede privada. Um melhor entendimento das constatações a que chegamos, que certamente refletem o que acontece no País, resultará em benefícios na abordagem terapêutica de quem padece dessa importante enfermidade.

A expressiva taxa de reinternações observada, que seguramente tem impacto significativo nos custos com saúde, decorre não só de fatores clínicos, mas também do tipo de assistência recebida pelo paciente, tanto durante a internação no hospital como após a alta. É oportuno salientar que cerca de 72% da população brasileira dependem exclusivamente do SUS. Por conseguinte, conforme ressaltamos, a Atenção Básica à Saúde (ABS) pode desempenhar papel crucial na adoção e no reforço de medidas de prevenção secundárias que seguramente lograrão êxito para quem padece particularmente de SCA. Segundo a Portaria no 2.436 do Ministério da Saúde, publicada em 21/9/2017, constitui diretriz da Política Nacional de Atenção Básica a longitudinalidade do cuidado, com prestação de acompanhamento dos efeitos das intervenções em saúde e coordenação do cuidado. Portanto, o cuidado centrado na pessoa, também defendido nessa portaria, faz com que a ABS exerça papel fundamental no auxílio dos pacientes para que desenvolvam conhecimentos, aptidões e competência para melhor cuidarem da própria saúde. As ações de educação em saúde são fundamentais para reforçar o autocuidado e a prevenção de reinternações subsequentes. Por fim, a corresponsabilização entre profissionais de saúde e pacientes na implementação de ações de prevenção de complicações da doença, promoção de saúde e adequada adesão terapêutica pode influir positivamente na redução das taxas de reinternação.

Atenciosamente,

Larissa Marina Santana Mendonça de Oliveira

Danielle Góes da Silva

Antônio Carlos Sobral Sousa
