# Supplementary material for: Resposta a ‘’Reinternação de Pacientes com Síndrome Coronariana Aguda e seus Determinantes’’: Uma Visão da APS
Source: Arq Bras Cardiol. 2020 Apr 6;114(3):574–5. [Article in Portuguese] doi: 10.36660/abc.20190817 (PMC7792732; doi:10.36660/abc.20190817)
Supplement: Translation

# Translation: Response to “Readmission of Patients with Acute Coronary Syndrome and its Determinants”: An Overview of PHC

**Authors:** Laís Cruz Lima, Eliane Mazzuco, André Luís Prudêncio

**Affiliations:** 1 BrazilUniversidade do Sul de Santa Catarina (UNISUL), Tubarão, SC – Brazil

**Keywords:** Primary Health Care, Hospitalization/economy, Patient Readmission/economy, Health Services, Acute Coronary Syndrome, Socioeconomics Factors

The study by Oliveira et al.^1^ shows extremely important data on readmissions of patients within one year after hospitalization for ACS and its determinants. In their study, a number of variables were evaluated. The most determinant one includes the type of health service used by patients during hospitalizations.

Thus, for a better understanding of the reality portrayed not only in the host city of the study, but in many other cities throughout Brazil, and in order to allow a greater understanding of the variables addressed, we would like to highlight issues previously discussed in the literature.

The article shows that, of 21.46% of the patients who required readmission, the majority used private health services, highlighting one of the risk factors used in the study: the socioeconomic profile of the participants. Among those who depended on the public health system, the readmission rate was lower, because, as the spots are limited, not all of them are admitted, as opposed to what is seen in the private service.

The study reports that the demand for hospital service is because it is difficult to make an appointment at the primary care level, evidencing failure in communication between primary health care and the population, which, in the long term, affects hospital admissions and their high — potentially avoidable — costs. According to Cecílio et al.,^2^ when it comes to the population’s access to health services, primary health care is the gateway to this system, highlighting one of the fundamental roles of basic health units: prevention, whether primary, secondary or tertiary.

For Starfield,^3^ it is Primary Health Care that must coordinate the flow of users between the multiple health services, in order to ensure greater equity in access to health services. However, this requires an informed and supported community that is encouraged to prevent cardiovascular or other diseases, both before and after hospitalizations. This reality can be made possible with campaigns, projects, tracking and monitoring by the health unit in charge in each region.

Thus, it is extremely important that health professionals inform the population about the services offered by Primary Health Care and its role in disease prevention and prevention of hospitalization. In doing so, they can provide an alternative potentially capable of reducing hospitalization rates by the public health service during and after the duration of ACS. This way, it will be possible to enforce Law 8080,^4^ of 19/9/1990, of the Brazilian Constitution, which aims to promote health for all.

## Reply

We are thankful to the comments and for the interest in our study, which is about a relevant topic, i.e., readmission of patients with acute coronary syndrome (ACS) and its determinants to both public hospitals (Brazil’s public health system — SUS) and private hospitals. A better understanding of the findings we reached, which certainly reflect what happens in the country, will result in benefits for the therapeutic approach of those who have this major disease.

The expressive rate of readmissions observed, which certainly has a significant impact on health costs, results not only from clinical factors, but also from the type of assistance received by the patient, both during hospitalization and after discharge. It is worth noting that about 72% of the Brazilian population depends exclusively on the public health system. Therefore, as we said, Primary Health Care (PHC) can play a pivotal role in the adoption and reinforcement of secondary prevention measures that will surely be successful for those particularly affected by ACS. According to Ordinance No. 2436 of the Ministry of Health, published on 21/9/2017, Brazil’s Policy on Primary Health Care provides for the longitudinality of care, with monitoring of the effects of health interventions and coordination of care. Therefore, person-centered care, also advocated in this ordinance, places PHC in a fundamental role of helping patients to develop knowledge, skills and competence to better take care of their own health. Health education actions are essential to stress self-care and the prevention of subsequent readmissions. Finally, co-responsibility between health professionals and patients in the implementation of actions to prevent complications of the disease, health promotion and proper therapeutic adherence can positively influence the reduction of readmission rates.

Sincerely,

Larissa Marina Santana Mendonça de Oliveira

Danielle Góes da Silva

Antônio Carlos Sobral Sousa
